# Evaluation of Injured Axons Using Two-Photon Excited Fluorescence Microscopy after Spinal Cord Contusion Injury in YFP-H Line Mice

**DOI:** 10.3390/ijms160715785

**Published:** 2015-07-10

**Authors:** Hideki Horiuchi, Yusuke Oshima, Tadanori Ogata, Tadao Morino, Seiji Matsuda, Hiromasa Miura, Takeshi Imamura

**Affiliations:** 1 Department of Orthopaedic Surgery, Ehime University Graduate School of Medicine, Ehime 791-0295, Japan; E-Mails: ogata@m.ehime-u.ac.jp (T.O.); morino@m.ehime-u.ac.jp (T.M.); miura@m.ehime-u.ac.jp (H.M.); 2 Department of Molecular Medicine for Pathogenesis, Ehime University Graduate School of Medicine, Ehime 791-0295, Japan; E-Mail: timamura-ind@umin.ac.jp; 3 Translational Research Center, Ehime University Hospital, Ehime 791-0295, Japan; 4 Division of Bio-imaging, Proteo-Science Center, Ehime University, Ehime 791-0295, Japan; 5 Department of Anatomy and Embryology, Ehime University Graduate School of Medicine, Ehime 791-0295, Japan; E-Mail: matsuda@m.ehime-u.ac.jp

**Keywords:** spinal cord injury, axon degeneration, multiphoton excitation microscope, transgenic mouse

## Abstract

Elucidation of the process of degeneration of injured axons is important for the development of therapeutic modules for the treatment of spinal cord injuries. The aim of this study was to establish a method for time-lapse observation of injured axons in living animals after spinal cord contusion injury. YFP (yellow fluorescent protein)-H transgenic mice, which we used in this study, express fluorescence in their nerve fibers. Contusion damage to the spinal cord at the 11th vertebra was performed by IH (Infinite Horizon) impactor, which applied a pressure of 50 kdyn. The damaged spinal cords were re-exposed during the observation period under anesthesia, and then observed by two-photon excited fluorescence microscopy, which can observe deep regions of tissues including spinal cord axons. No significant morphological change of injured axons was observed immediately after injury. Three days after injury, the number of axons decreased, and residual axons were fragmented. Seven days after injury, only fragments were present in the damaged tissue. No hind-limb movement was observed during the observation period after injury. Despite the immediate paresis of hind-limbs following the contusion injury, the morphological degeneration of injured axons was delayed. This method may help clarification of pathophysiology of axon degeneration and development of therapeutic modules for the treatment of spinal cord injury.

## 1. Introduction

Fluorescence microscopy techniques have been developed to enable observation of deep tissue. Confocal microscopy excludes out-of-focus information and provides images of deep regions of thick samples [1]. The most recent inventions in this field are multiphoton excitation methods, first demonstrated by Denk *et al.* in 1990 [2]. Those authors used two-photon excited fluorescence imaging to generate an optical section of live cultured pig kidney cells stained with Hoechst 33258. Use of infrared lasers for two-photon excitation of chromophores, which are ordinarily excited with visible light, may prevent the usual photodynamic perturbation and facilitate protracted fluorescence observations of living cells. Victria *et al.* described the advantages of multiphoton excitation methods, relative to imaging by confocal microscopy, for observation of deeper tissue [3].

These methods have elucidated the pathophysiology of several diseases. Christie *et al.* reported that senile plaques in Tg2576 mice stained with isoflavine S could be clearly visualized using *in vivo* multiphoton laser scanning microscopy [4]. Kienast *et al.* observed the relationship between fluorescence-labeled cancer cells and perfused blood vessels using a multiphoton laser-scanning microscope through a chronic cranial window over a period of minutes to months, up to a depth of 500 μm in the live brain of nude mice [5]. Carson *et al.* observed the location of emboli using two-photon microscopy after internal carotid infusion of fluorescently conjugated microemboli in living mice [6].

The multiphoton excitation method requires clearly visualized tissue fluorescence. Yellow fluorescent protein (YFP)-expressing mice (YFP-H line) [7], which were used in this study, exhibit fluorescence in most neuronal tissue, including neuronal cell bodies, axons, and dendrites. Kawakami *et al.* reported that cerebral neocortex tissue could be clearly visualized in a living mouse using a superior tissue penetration method with two-photon microscopy [8]. Enhanced YFP (EYFP) fluorescence can be detected from layers deeper than 1.4 mm beneath the brain surface in an anaesthetized mouse. Moreover, three dimensional reconstruction of individual neural cell spreading in layers I–V is feasible without any degradation of submicron spatial resolution. These mice have been used in several key studies: using YFP mice, Charles *et al.* observed nerve filaments distributed in the cornea [9]; Beirowski *et al.* observed Wallerian degeneration processes in L4 spinal nerves after resection [10]; and Carter *et al.* demonstrated that Chondroitinase ABC treatment prevented axonal degeneration and promoted neuronal regeneration after crushing of the dorsal column [11].

In order to develop therapeutic modules for the treatment of spinal cord injury, it is necessary to observe degeneration processes. After traumatic spinal cord injury, primary damage such as bleeding and axonal tearing is followed by secondary degeneration of damaged neural tissue. Secondary degeneration is believed to be produced by ischemia, tissue edema, glutamate-induced excitotoxicity, or elevation of intracellular calcium [12]. Apoptotic cell death also plays a role in this secondary degeneration [13]. The spinal cord consists of gray matter, which includes neuronal cell bodies, and of white matter, which primarily includes axons and myelin structures. In particular, pyramidal tracts from central motor neurons in the cerebral cortex to spinal motor neurons are the most important organs involved in the onset of paresis. In rats and mice, in contrast to humans, pyramidal tracts exist in the dorsal funiculi. Therefore, it is important to observe the degeneration processes of axons in dorsal funiculi after spinal cord injury. We previously established *in vivo* imaging of axon fibers in dorsal funiculi using a compact Ytterbium (Yb)-fiber laser at 1045 nm for multiphoton excited fluorescence microscopy [14]. Farrar *et al.* observed the degeneration of axons after a laser-induced spinal cord injury in YFP-H mice using two-photon excited fluorescence microscopy [15]. However, actual traumatic spinal cord injuries are usually caused by contusion damage. Therefore, observation of spinal cord tissue after contusion injury is necessary for an understanding of cellular dynamics and for development of treatment after traumatic spinal cord injury.

The aim of this study was to establish a time-lapse observation system for the evaluation of axon degeneration after spinal cord injury. In order to produce conditions similar to clinical situations, we employed a contusion injury model. We also tried to observe real-time axon degeneration processes in living animals.

## 2. Results

**Figure 1:**
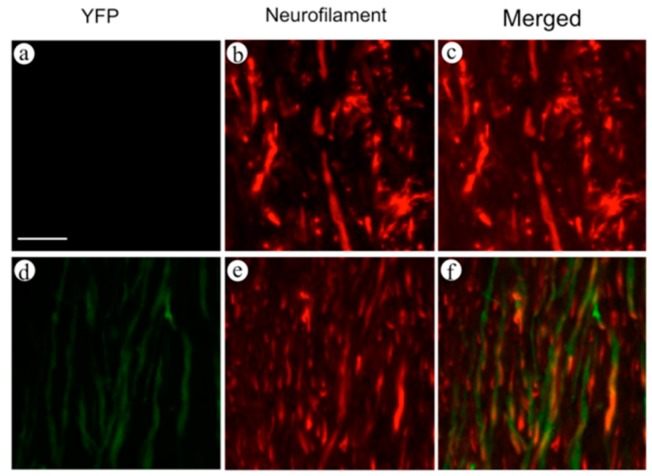
Double staining of neurofilaments and YFP in the spinal cord of both wild-type (**a**–**c**) and YFP-H mice (**d**–**f**). Observation was performed using conventional fluorescence microscopy. Expression of YFP was labeled with FITC (**a**,**d**), and neurofilaments were labeled with Cy3 (**b**,**e**). Merged images were **c** and **f**. Scale bar is 25 μm.

In order to confirm the expression of yellow fluorescent protein (YFP) on axons, we performed double staining of neurofilaments and YFP in the spinal cord in both wild-type and YFP-H mice. Observation was performed by conventional fluorescence microscopy (Figure 1). In the wild-type mice (Figure 1a–c), no fluorescent signal was observed (Figure 1a). On the other hand, fibers with a clear fluorescent signal, representing YFP, were observed in YFP-H mice (Figure 1d). Fibers positive for anti-neurofilament antibodies were clearly observed in both wild-type (Figure 1b) and YFP-H mice (Figure 1e). In merged images, most of the YFP-positive fibers overlapped with the neurofilament-positive axons in YFP-H mice (Figure 1f).

**Figure 2:**
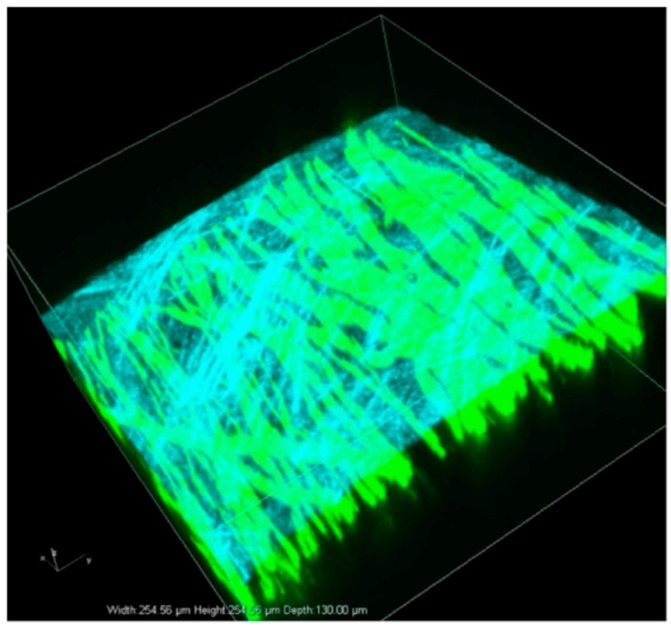
Intact normal axons in the dorsal funiculi of YFP H-line mice were observed by two-photon excitation microscopy. Blue color represents the SHG signal from the dura, which covered the surface of the spinal cord. The green signal represents the YFP fluorescence signal. Image acquisition was performed in an area of 254.5 × 254.5 μm at seconds per frame. A three-dimensional volume-rendering image was constructed using the z-stack images.

Figure 2 shows a three-dimensional rendering image of axons in dorsal funiculi in a living YFP-H mouse. Using two-photon excitation microscopy, fibers with a YFP signal were observed up to ~150 μm in depth from the surface of the spinal cord. Because collagen fibers have a non-centrosymmetric structure, we could also observe collagen structures as second harmonic generation (SHG) signals (blue signal in Figure 2) in the dura mater covering the spinal cord.

**Figure 3:**
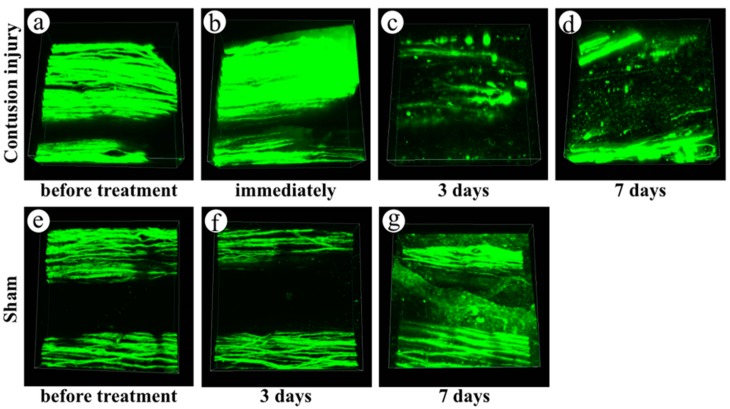
Time-course change of dorsal funiculi axons in YFP-H mice after spinal contusion injury, observed using two-photon excitation microscopy. Spinal cord contusion injury mice were observed before treatment (**a**) and immediately (**b**), three days (**c**), and seven days (**d**) after treatment; sham mice were observed before treatment (**e**), three days (**f**), and seven days (**g**) after treatment. Image acquisition was performed in an area of 509 × 509 μm at seconds per frame. A three-dimensional volume-rendering image was constructed using the z-stack images. Left side is cranial, and right side is caudal.

We then observed the change in dorsal funiculi axons in the center of injury in YFP-H mice over a time course using two-photon excitation microscopy (Figure 3). Specifically, we performed time-lapse observation of dorsal funiculi in animals that had undergone spinal cord contusion injury (Figure 3a–d). Intact axonal fibers in dorsal funiculi were observed before injury (Figure 3a). When we observed the dorsal spinal cord immediately after contusion injury, intact axon structures were still present (Figure 3b). Three days after injury, the spinal cord was swollen, and some axons had fragmented (Figure 3c). Seven days after injury, the extent of axon fragmentation had increased (Figure 3d). In the sham animals that underwent removal of lamina without contusion injury, intact axon fibers without degeneration were observed before treatment (Figure 3e), at three days (Figure 3f), and at seven days (Figure 3g) after the sham operation. Thickening of dura with fibrosis occurred over time due to repetition of the operation. Figure 3g shows the blurred signaling resulting from the thickened dura mater in a sham mouse seven days after the operation. Despite this blurring, fine structures of axons could still be observed.

**Figure 4:**
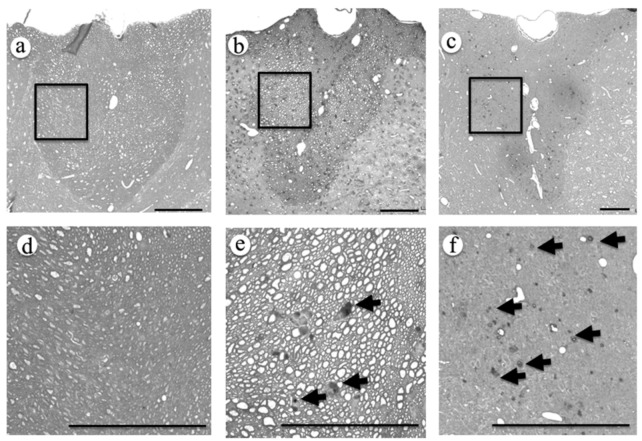
Dorsal funiculi of spinal cord before (**a**,**d**), three days (**b**,**e**), and seven days (**c**,**f**) after contusion injury. Images (**d**–**f**) were high magnification images of areas boxed in (**a**–**c**), respectively. Degenerated nerve fibers (arrows) were observed in dorsal funiculi three days and seven days after injury. Scale bar is 100 μm.

Axon degeneration was also evaluated by osmium staining, a conventional method for identification of degenerated axons. Before contusion injury, spinal cord sections contained few osmiophilic degenerated axons (Figure 4a). Three days after contusion injury, many osmiophilic degenerated axons were clearly detectable in the dorsal funiculi (Figure 4b). Seven days after injury, the number of degenerated axons in the dorsal funiculi rose further (Figure 4c).

**Figure 5:**
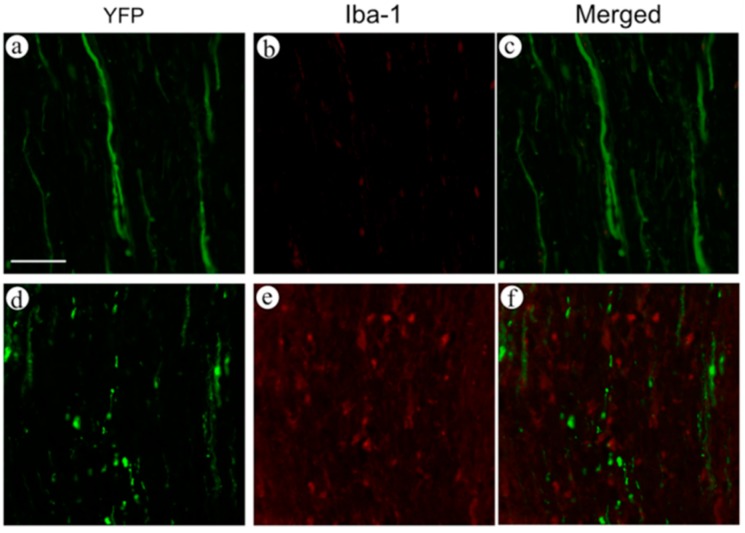
Double staining of YFP and Iba-1, a marker of microglia and macrophages, in the spinal cord of both normal (**a**–**c**) and contusion injury mice (**d**–**f**) seven days after treatment. YFP was labeled with FITC (**a**,**d**). Iba-1 was labeled with Cy3 (**b**,**e**). Merged images are shown in **c** and **f**. Scale bar is 50 μm.

To evaluate the relationship between axonal degeneration and the inflammatory response, we performed double staining of spinal cord sections using FITC-labeled anti-GFP antibodies and Cy3-labelled anti-Iba-1 antibodies, in both normal and contusion injury models in YFP-H mice, seven days after treatment. Observation was performed by conventional fluorescence microscopy (Figure 5). In dorsal funiculi of normal mice (Figure 5a–c), the continuity of YFP-positive fibers was maintained (Figure 5a), and few Iba-1-positive cells were present (Figure 5b). In mice that had undergone spinal cord contusion injury, we observed fragmentation of YFP-positive fibers, similar to the axonal change we observed by two-photon excitation microscopy (Figure 5d). Large numbers of Iba-1-positive cells were present in the dorsal funiculi (Figure 5e). In merged images, Iba-1-positive cells surrounded the fragmented YFP-positive fibers (Figure 5f).

**Figure 6:**
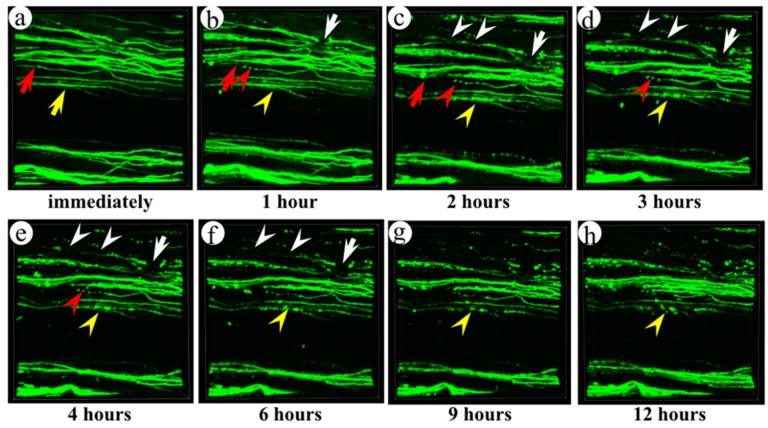
Continuous observation of axon degeneration in the early phase after spinal cord contusion injury using two-photon excitation microscopy. YFP-positive fibers in the injured spinal cord were observed immediately (**a**), 1 h (**b**), 2 h (**c**), 3 h (**d**), 4 h (**e**), 6 h (**f**), 9 h (**g**), and 12 h (**h**) after the injury. The initial contusive injury occurred immediately (see white arrows and red arrows) and then was followed by axonal fragmentation (white and red arrowheads). An injured axon could also become swollen 1–2 h after the injury and then become fragmented (yellow arrowheads). Image acquisition was performed in an area of 509 × 509 μm at seconds per frame. A three-dimensional volume-rendering image was constructed using the z-stack images. Left side is cranial, and right side is caudal.

In order to evaluate acute axon degeneration, we performed continuous observation of individual injured axons in YFP-H mice using two-photon excitation microscopy at an early period (<12 h) after the injury. The mouse, in which the thoracic spinal cord was exposed by removing the lamina, was fixed on the stage of the microscope under general anesthesia until 12 h after the injury. The axon, which was injured by the initial contusive damage (see white arrows in Figure 6a), still maintained its structure at an hour after the injury (Figure 6b). In this axon, fragmentation had occurred at 2 to 3 h after the injury (white arrowheads in Figure 6c,d). The axon lost its continuity at 4 to 6 h after the injury (white arrowheads in Figure 6e,f) and disappeared at 9 h after the injury (Figure 6g). This fragmentation could be bi-directional, including Wallerian degeneration of distal portion of axons and axonal die-back of proximal portions. Typical Wallerian degeneration was also observed in this experiment (red arrow in Figure 6a). The degeneration progressed from the proximal to distal direction (red arrowheads in Figure 6b–e). The axons, which had appeared intact immediately after the injury (yellow arrow in Figure 6a), began to swell an hour after the injury (Figure 6b). Apparent fragmentation in this filament was observed at 3 to 4 h after the injury (see yellow arrowheads in Figure 6d,e). Then, axon continuity disappeared at 9 h after the injury (yellow arrowheads in Figure 6g). On the other hand, some YFP-positive fibers without fragmentation were present at 12 h after the injury.

## 3. Discussion

In this study, we analyzed time-course morphological changes of axon degeneration after a traumatic spinal cord injury that resembles actual clinical injuries. For this purpose, we established an experimental spinal cord contusion injury model and observed injured axons by two-photon excitation fluorescence microscopy, which is widely used for examination of deep tissues in living animals [8]. Two-photon excitation fluorescence microscopy also enabled us to visualize the dura, which contains a substantial amount of collagen fibers, by SHG without fluorescence labeling or staining [14,15]. Figure 2 shows the relationship between the axons in the dorsal funiculi and collagen fibers in the dura mater. SHG signaling increased with time after contusion injury (data not shown), possibly due to hypertrophy and fibrosis of the dura mater following mechanical damage. In this research, we focused on the change in YFP signaling; therefore, only one image is shown in Figure 2.

In YFP-H mice, YFP signals are believed to represent nervous tissue such as neuronal soma, axons, and dendrites [7]. In order to confirm that the YFP signals in the dorsal funiculi are expressed in axons, we performed double staining for YFP and neurofilaments (Figure 1). Most fibers that expressed YFP signals overlapped with the fibers positive for anti-neurofilament antibodies. We convinced ourselves that YFP-positive fibers in the dorsal funiculi represented axons in the white matter. The results of osmium staining, which recognizes degenerated axons (Figure 4), were quite similar to results obtained by observing fragmentation of YFP-positive fibers (Figure 3). Thus, we conclude that fragmentation of the YFP signal is the result of the degeneration of injured axons.

One of the limitations of our research is that we observed only a center of the injured part. The axon degeneration should also have occurred at the distal and proximal part from the injured center. In order to minimize the invasion of the operation, we performed the laminectomy in a small area which was the necessary minimum for the observation of the impact center. If we performed a wider laminectomy, we could also have observed the distal and proximal part from the injured center. However, a wider laminectomy increases risks such as hemorrhage or infection when we perform a repeated operation. Some new devices to avoide the disadvantage were required. Fenrich *et al.* reported a simple glass window methodology for chronic *in vivo* spinal cord microscopy [16,17]. Using these devices, it may be possible to observe a wide area of the spinal cord without repeated surgical procedures.

In this experiment, we observed axon degeneration from the dorsal surface of the dorsal funiculi and YFP-positive fibers were observed up to ~150 μm in depth from the surface of the spinal cord tissue using two-photon excitation microscopy, although YFP-positive fibers were detected from layers deeper than 1.4 mm beneath the brain surface in the transgenic mice [8]. These results were due to the difference of optical properties such as light scattering and absorption between brain and spinal cord tissue. In the spinal cord, the penetration depth is not so large when compared to the brain. Therefore, axons of sensory nerves could be clearly shown by our method. Quintá *et al.* reported that the corticospinal tract labeled with anterograde tracers in the spinal cord was observed using one-photon confocal microscopy in the excised and fixed tissue of mice [18]. The distance from the dorsal surface of the spinal cord to the corticospinal tract was less than 150 μm. Some corticospinal tracts might be seen in parallel with sensory axons in our study. Bareyre *et al.* reported that the corticospinal tract (CST) fibers are specifically and completely labeled by YFP in transgenic mice (CST-YFP mice) [19]. In the future, the observation specializing in motor axons will be possible in this established method if we employ CST-YFP mice. Our experimental system will also be useful to focus on some individual injured axons to observe the degeneration process *in vivo*. In the current observation system, it was technically difficult to identify each individual injured axon. We previously reported the development of the laser ablation system combined with the multiphoton microscope [14]. In the next step, individual axons should be examined once our microscopic techniques are improved.

To assess the relationship between neuronal degeneration and immune reactions in glial cells, we performed double staining of inflammatory cells, such as microglia and macrophages (Iba-1-positive) and neuronal fibers (YFP signal), and observed them by conventional fluorescence microscopy. The Iba-1-positive cells are mixture of microglia and macrophages. After the traumatic central nervous system (CNS) damage, microglia in the CNS proliferated and macrophages migrated from vessels to the inflammation in the damaged area. Delayed degeneration of axons, presented in this study, is believed to be induced by inflammatory responses in damaged neuronal tissue. Microglia, which are immune cells within the central nervous system, proliferate in many neurodegenerative diseases, such as Alzheimer’s disease [20], Parkinson’s disease [21,22], and HIV encephalopathy [23,24]. In addition, proliferation and activation of microglia in the injured spinal cord has been demonstrated in experimental spinal cord injury models [25,26]. Pathologically activated microglial cells release various harmful components such as nitric oxide (NO), superoxide (O_2_^−^), and several kinds of cytokines, including interleukins (IL)-1, IL-6, and tumor necrosis factor α (TNF-α), which can directly or indirectly affect the biological functions of neurons, aggravate neurotoxicity, and may even exacerbate neurodegeneration and cell death [27,28,29,30,31,32]. In this study (Figure 5), we observed large numbers of Iba-1–positive cells in the dorsal funiculi in injured animals, but not normal animals. In other words, the proliferation of microglia and the migration of macrophages in the dorsal funiculi were caused by contusion injury. The fragmented YFP-positive filaments were surrounded by proliferated Iba-1–positive cells. Thus, it is possible that proliferated and activated inflammatory cells induced delayed axonal degeneration (fragmentation) in the injured dorsal funiculi.

Axon degeneration in the acute phase (up to 12 h) was continuously observed under general anesthesia (Figure 6). Even in the axons, which seemed to be intact immediately after the injury, degeneration processes progressed from the swelling (1 to 2 h) to fragmentation (3 to 6 h in Figure 6, yellow arrow and arrowheads). The axons lost their continuity at 9 h after the injury. This delayed degeneration without the mechanical tear of axons may be induced by the toxic substances released from the surrounding glial cells. Additionally, it has been reported that microglia release cytokines and nitric oxides which induced apoptosis of neurons [27,28,29]. In this experiment, we observed remarkable microglia proliferation in the injured spinal cord tissue (Figure 5). This proliferated microglia might induce swelling following the fragmentation of axons which did not get injured by mechanical force.

The mice used in our injury model, which suffered thoracic spinal cord injuries caused by a pressure of 50 kdyn, exhibited complete paresis of their hind-limbs immediately after injury. However, many morphologically intact axons remained in the injured part at that time (Figure 6). Two hours after the injuries, some remaining axons had begun to degenerate. Subsequently, the number of fragmented axons increased with time. However, some YFP-positive fibers without fragmentation were present at 12 h after injury. This observation indicates that axonal degeneration occurs not only in an acute phase, but also in some delayed processes after spinal cord injury. Apparent conduction depression of the cortico-spinal tract occurred immediately after injury, despite the preservation of intact axons in the dorsal funiculi. Although it is not clear why motor signals were disrupted immediately after the injury, this finding suggests that we have a chance to treat the injured spinal cord to prevent secondary degeneration of axons after spinal cord injury. It is possible that some therapies (hypothermia or p38 MAPK inhibitor) can be applied before an irreversible morphological change of the axons takes place [33,34].

## 4. Experimental Section

### 4.1. Animals

YFP-H transgenic mice (B6.Cg-Tg(Thy1-YFP)H2Jrs/J, The Jackson Laboratory, Bar Harbor, ME, USA) were used for this study [7]. The research protocol was accepted by the Ethical Committee for Animal Experiments of Ehime University (approval ID: #05-RE1-16, approval date: 10 June 2011).

### 4.2. Spinal Cord Injury Model

Mouse spinal cords were carefully exposed by removal of the vertebral lamina at the 11th vertebra under general anesthesia with isoflurane. Spinal cord injury was performed using IH impactor (MUROMACHI KIKAI CO., LTD., Tokyo, Japan), which applied a pressure of 50 kdyn [35]. In this model, no hind-limb movement was observed in most mice during the observation period. In sham-treated mice, removing of lamina was performed without spinal cord injury.

### 4.3. Two-Photon Excitation Fluorescence Microscopy

We have established a method for *in vivo* imaging of spinal cord injury in mice [14]. In brief, for microscopic observation, the spinal cord of YFP-H mice was exposed under general anesthesia with isoflurane, and axons were visualized using an upright two-photon excitation fluorescence microscopy system (A1RMP, Nikon Corporation, Tokyo, Japan). The vertebrae were fixed by clamping to the stage of the microscope. We performed two-photon excited fluorescence imaging with a 25× water-immersion objective lens (CFI75 Apo 25× W MP, numerical aperture = 1.1; Nikon) and excitation at 950 nm, 80 MHz repetition rate, and 70 fs pulse width from a Ti:sapphire laser oscillator (MaiTai eHP, Spectra-Physics, Santa Clara, CA, USA). We employed an emission filter at 525/50 nm (center wavelength/bandwidth) to isolate the fluorescence signal from EYFP, and used a short pass filter at 492 nm to obtain a second harmonic generation (SHG) signal from the dura mater of the spinal cord [36]. Image acquisition and processing for three-dimensional rendering was performed using NIS-Elements ver. 4.1 (Nikon). Each image was taken in galvano scanner mode (1 frame per second, 512 × 512 pixels) from the surface of the dorsal cord to the deep portion (approximately 150 μm), and the images were stacked in the depth direction (z-stack image acquisition). The laser power and gain of the detector were adjusted according to the signal level each of the channels (SHG and YFP). We observed the spinal cord of the same mouse before and after injury by two-photon excitation microscopy in the manner described above.

### 4.4. Histological Examination

Mice were sacrificed for histological study by cervical dislocation under deep anesthesia with isoflurane, and their spinal cords at the 11th vertebral level (point of contusion) were immediately removed. Horizontal or axial frozen sections with a thickness of 10 μm were produced. To confirm the expression of YFP on axons at the normal spinal cord, some sections were subjected to double immunostaining of neurofilaments and YFP. The sections were fixed on glass slides with 4% paraformaldehyde in phosphate-buffered saline (PBS) for 5 min. Then, after two washes with PBS, the slices on the slides were exposed to anti-neurofilament (1:1000 in 1% horse serum/PBS; Cell Signaling Technology Japan, K.K. Tokyo, Japan) and anti-GFP (1:1000 in 1% horse serum/PBS; Medical and Biological Laboratories Co., Ltd., Nagoya, Japan) overnight at 4 °C. Then, after two washes with PBS for 10 min each, slides were exposed at room temperature for 60 min to the secondary antibody: Cy3-conjugated anti-mouse IgG (1:100 in 1% 1% horse serum; GeneTex, Irvine, CA, USA) for neurofilaments, and FITC-conjugated anti-rabbit IgG (1:80 in 1% horse serum/PBS; Sigma-Aldrich Co. LLC., St. Louis, MO, USA) for GFP. After two washes with PBS, the sections were observed under a wide-field upright fluorescence microscope (ECLIPSE 80i, Nikon).

To determine the relationship between axonal degeneration and inflammation, double staining of YFP/ Iba-1 was performed in a manner similar to that described above. For double staining of Iba-1 with YFP, slices on slides were incubated overnight at 4 °C with the appropriate antibodies, *i.e.*, anti-Iba-1 antibody (1:500 in 1% BSA/TBST; Abcam, Tokyo, Japan) or anti-GFP antibody (1:1000 in 1% BSA/TBST; Medical and Biological Laboratories, Nagoya, Japan). The sections were further stained with secondary antibodies and observed as described above.

In order to identify degenerated axons, some sections were stained post-fixed in osmic acid using saturated aqueous uranyl acetate and embedded in EPON epoxy resin as described previously [37]. Briefly, mice were perfused transcardially with a fixative solution containing 4% paraformaldehyde and 2% glutaraldehyde in 0.1 M phosphate buffer. The spinal cord was removed, sliced transversely, and post-fixed in 4% osmic acid. The tissue was stained *en bloc* using saturated aqueous uranyl acetate solution for 10 h, and then dehydrated through a graded ethanol series and embedded in EPON epoxy resin. The tissue was then cut into 0.5-μm thick transverse sections and stained using toluidine blue. In these histological sections, the osmiophilic degenerated axonal debris stained black.

## 5. Conclusions

In this study, we established a method for time-lapse examinations of axon degeneration after contusion injury of the spinal cord, using EYFP H-line mice and two-photon excitation fluorescence microscopy. Using this method, we will be able to understand molecular mechanisms and cellular dynamics in response to traumatic spinal cord injury. This system, which we demonstrated in this study, may also be useful for the evaluation of novel neuroprotective modules. We are also very interested in determining the effective window for treatments following injury. Detailed information about neurodegenerative processes, including their time course, may accelerate the development of therapeutic methods for the treatment of traumatic spinal cord injury.
